# 36% Enhanced Efficiency of Ternary Organic Solar Cells by Doping a NT-Based Polymer as an Electron-Cascade Donor

**DOI:** 10.3390/polym10070703

**Published:** 2018-06-25

**Authors:** Jianfeng Li, Zezhou Liang, Yichun Peng, Jie Lv, Xuying Ma, Yufei Wang, Yangjun Xia

**Affiliations:** 1 Key Laboratory of Optoelectronic Technology and Intelligent Control of Education Ministry, Lanzhou Jiaotong University, Lanzhou 730070, China; zezhouliang@gmail.com (Z.L.); zoeyejie@163.com (J.L.); maxuying2016@126.com (X.M.); yufeiwang96@163.com (Y.W.); 2 School of Civil Engineering, Lanzhou Institute of Technology, Lanzhou 730050, China; pycljf@163.com

**Keywords:** ternary organic photovoltaic cells, complementary absorption, cascade energy levels, PTT-DTNT-DT

## Abstract

In recent years, ternary organic photovoltaic cells (OPVs) have been dedicated to improving power conversion efficiency (PCE) by broadening optical absorption spectra. Ternary OPVs with different poly[thieno[3,2-b]thiophene-2,5-diyl-alt-4,9-bis(4-(2-decyltetradecyl)thien-2-yl)naphtho[1,2-c:5,6-c’]bis[1,2,5]thiadiazole-5,5′-diyl] (PTT-DTNT-DT) doping concentrations were designed and the effect of PTT-DTNT-DT as a complementary electron donor on the performance of OPVs was investigated. The optimized PCE of OPVs was increased from 3.42% to 4.66% by doping 20 wt % PTT-DTNT-DT. The remarkable improvement in the performance of the ternary device is mainly attributed to the sharp increase in the short-circuit current density and fill-factor. The major reasons have been systematically studied from atomic force microscopy, electrochemical impedance spectroscopy, surface energy, space charge limited current and photocurrent behavior. It has been found that the separation of excitons and the transportation of charge are enhanced while light absorption is increased, and the charge recombination also decreases due to the optimization of the cascade energy level and the morphology of the ternary active layer. The results show that it is feasible to improve the performance of ternary OPVs by their complementary absorption.

## 1. Introduction

Bulk heterojunction (BHJ) organic photovoltaic cells (OPVs) have been widely developed for their advantages of low cost, low environmental pollution, simple process and large area preparation [1,2,3]. Many strategies have been devised to elevate OPVs performance; for example, designing new device structures [4], designing and synthesizing high-performance materials [5,6], using effective interlayers [7], and optimizing device morphology [8,9]. Although the power conversion efficiency (PCE) has been greatly improved in the recent decade, it is still restricted by a few factors, such as insufficient optical absorption and low carrier mobility [10,11]. Ternary strategy has emerged as an effective technique to not only improve photon capture and charge carrier mobility, but also maintain a simple manufacturing process for single-layer active devices, rather than more complex tandem cells [12]. For ternary blend OPVs with “two donors/one acceptor”, the third component should be a material whose absorption spectral is complementary with that of the host system; this was helpful to improve the photonic capture performance of active layers with different bandgap materials [13,14,15,16,17,18,19,20,21,22].

In order to supplement the absorption spectrum, the focus of attention has been the study of additional donors with near-infrared absorption in ternary OPVs. The low bandgap polymers are normally doped in the P3HT:PC_61_BM system, and the enhanced short-circuit current density (*J*_SC_) and thus increased PCE [23,24,25]. A low bandgap polymer material poly[thieno[3,2-b] thiophene-2,5-diyl-alt-4,9-bis(4-(2-decyltetradecyl)-thien-2-yl) naphtho[1,2-c:5,6-c’]bis[1,2,5]thiadiazole-5,5′-diyl] (PTT-DTNT-DT) [26] not only exhibited a broad absorption to infrared region and was complementary to P3HT absorption spectrum, but also possessed cascade energy levels, which may benefit separation and transportation of charge [27]. Therefore, the PTT-DTNT-DT was considered to be the ideal complementary donor material in ternary OPVs.

In this work, the low bandgap polymer PTT-DTNT-DT was chosen as the second donor to incorporate into the P3HT and PC_61_BM binary active layer of OPVs to enhance the absorption of the blend. The OPVs were fabricated with the inverted configuration of ITO/PFN/active layer/MoO_3_/Ag. As a consequence, we obtained a PCE as high as 4.66% by doping 20 wt % PTT-DTNT-DT content compared with 3.42% PCE of binary cells. To investigate the mechanism of ternary PSCs doped PTT-DTNT-DT, the active layer morphology was characterized by atomic force microscopy (AFM) and the position of PTT-DTNT-DT in P3HT:PC_61_BM blends was studied by surface energy analysis. Besides, the impedance spectra were also analyzed in order to provide necessary information to research the electrical processes of ternary blends. Performance improvement was attributed to not only increased optical absorption and optimized device morphology, but also the facilitated charge separation and transportation by cascade energy levels.

## 2. Materials and Methods

### 2.1. Devices Fabrication

poly(3-hexylthiophene) (P3HT, *M*_w_ = 48,300 g·mol^−1^, 99% purity) and [6,6]-phenyl-C_60_-butyric acid methyl ester (PC_61_BM, 99.5% purity) were purchased from Sigma-Aldrich Co. (Oakville, ON, Canada), MoO_3_ (99.99%) and Ag (99.99%) were purchased from Alfa (Zhengzhou, China), and PTT-DTNT-DT was synthesized according to the literature [26]. The inverted configuration with ITO/PFN/Active Layer/MoO_3_/Ag was fabricated. The ITO (indium tin oxide) substrates were washed continuously with acetone, detergent, de-ionized water and isopropanol, dry and then treated under ultraviolet light for 3 h. A thin film of PFN was spin-coated (2400 rpm 40 s) on ITO substrate. Then the ternary blend solution of P3HT:PTT-DTNT-DT:PC_61_BM in orth-dichlorobenzene solvent (30 mg·mL^−1^) was spin-coated on the PFN layer at a speed of 600 rounds per minute for 30 s. Next, the samples were annealed on a hot plate at 135 °C for 20 min. Finally, about 8 nm MoO_3_ and 100 nm Ag were deposited by continuous evaporation under vacuum degree of 5 × 10^−4^ Pa. The active area of every device was 10 mm^2^.

### 2.2. Measurements and Characterizations

The absorption of P3HT and PTT-DTNT-DT were measured by a UV-1800 spectrophotometer (Shimadzu Co., Kyoto, Japan). The current density-voltage (*J*-*V*) curves were measured through a computer controlled by a Keithley 2400 source meter. The external quantum efficiency (EQE) of the devices were determined with an incident photon to charge carrier efficiency (IPCE) setup (7-SCSpecIII, Beijing 7-Star Optical Instruments Co., Beijing, China). The morphologies were observed by using an atomic force microscope (Asylum Research, MFP-3D-SA, Santa Barbara, CA, USA). The electrochemical impedance measurements were measured using an electrochemical workstation (CHI660E, Shanghai Chenhua, Shanghai, China). The contact angle was obtained by using a contact angle instrument (Kruss, model DSA100, Hamburg, Germany).

## 3. Results and Discussion

### 3.1. Photovoltaic Characteristics

**Figure 1:**
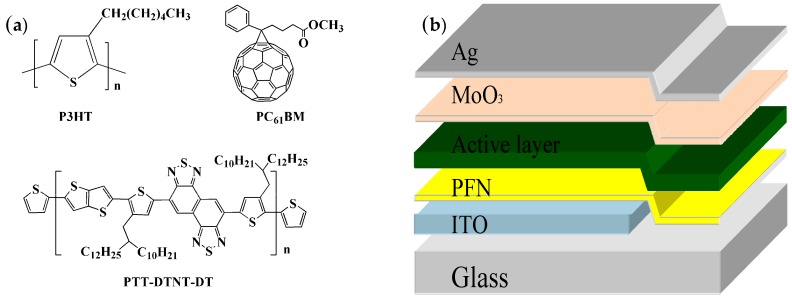
(**a**) Chemical structures of materials in active layer; (**b**) Device configuration of ternary OPVs.

**Figure 2:**
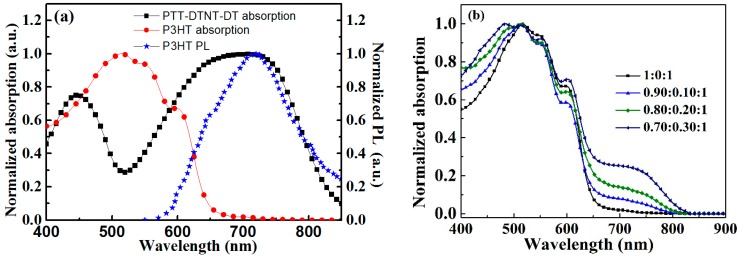
(**a**) Absorption of PTT-DTNT-DT film, Absorption and emission spectra of P3HT films; (**b**) Absorption of the blend films with different P3HT:PTT-DTNT-DT:PC_61_BM weight ratios.

The chemical structures of the used materials, P3HT, PTT-DTNT-DT and PC_61_BM, are shown in Figure 1a, and the inverted device structure is shown in Figure 1b. The absorption of P3HT and PTT-DTNT-DT are presented in Figure 2a. According to the UV-Vis absorption spectra, we can obtain that the absorption peak for P3HT was at 510 nm and the maximum absorption appeared at around 650–750 nm for PTT-DTNT-DT. The absorption of the ternary blend with PTT-DTNT-DT of various concentrations is shown in Figure 2b. It is clear that when doping PTT-DTNT-DT into P3HT:PC_61_BM, the optical absorption of the ternary blend was evidently widened to 840 nm and obviously enhanced by increasing the doping concentration.

**Figure 3:**
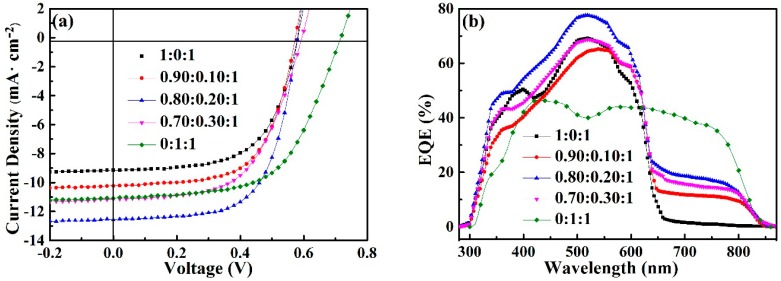
(**a**) Current-voltage curves of the binary and ternary devices; (**b**) the corresponding EQE spectra of the OPVs.

A series of OPVs with different P3HT:PTT-DTNT-DT:PC_61_BM weight ratios were fabricated under the same process condition. The corresponding *J-V* curves and performance parameter of all OPVs are exhibited in Figure 3a and Table 1, respectively. It is easy to see that the *J*_SC_ and *FF* increase first and then decrease with the increase of PTT-DTNT-DT doping concentration. *J*_SC_ increased to 12.58 mA·cm^−2^ by adding 20% PTT-DTNT-DT content, resulting in a PCE of 4.66%. When further increasing the third component beyond 20% content, we obtained degenerative performances with inferior *FF* compared with reference devices based on P3HT:PC_61_BM binary blend.

In order to in depth probe into the changes in *J*_SC_, the external quantum efficiency (EQE) of OPVs was measured and showed in Figure 3b. The integrated current density *J*_SC_ from EQE spectra for devices with the incorporation of 0%, 10%, 20% and 30% PTT-DTNT-DT were 9.62, 10.19, 12.68 and 11.21 mA·cm^−2^, respectively. These are in good agreement with those obtained by *J-V* measurement. It is clear that P3HT:PC_61_BM-based cells represent a relatively narrow response range (300–650 nm) compared with the EQE spectra (300–840 nm) of ternary devices. With the increasing of PTT-DTNT-DT doping concentration, EQE first increases and then decreases. The enhancements can be attributed to the improved photon capture and the matrix of cascade energy levels, which are beneficial to effective excitons separation and charge transportation in the ternary OPVs. With the further increase of PTT-DTNT-DT doping concentration, the *J*_SC_ and *FF* of ternary OPV decrease, which may have mainly resulted from the disrupted interpenetrated networks and the raised charge carrier recombination, which was in accordance with the exciton dissociation rate and atomic force microscope image analysis.

### 3.2. Photocurrent Behavior

**Figure 4:**
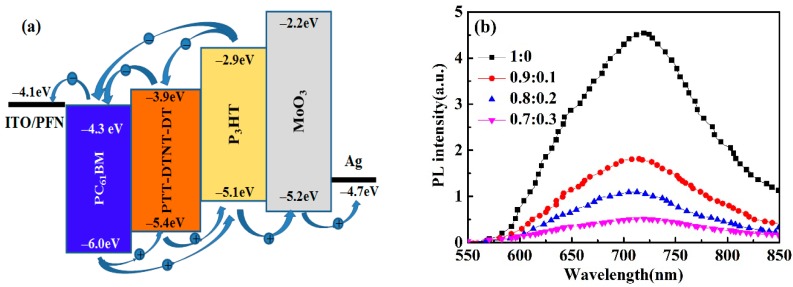
(**a**) Schematic diagram of energy level structure of ternary OPVs; (**b**) PL spectra of different proportion P3HT:PTT-DTNT-DT blend membranes.

The HOMO energy level of P3HT, PTT-DTNT-DT and PC_61_BM are −5.1, −5.38 [26] and −6.0 eV. Meanwhile, the LUMO energy levels of these materials are −2.9 eV, −3.90 eV [26] and −4.28 eV, respectively. As you can see from Figure 4a, the HOMO and LUMO levels of PTT-DTNT-DT are just between the homologous energy levels of P3HT and PC_61_BM. This cascade energy level structure can provide more effective paths for exciton separation and charge carrier transport at the interfaces of P3HT/PC_61_BM, PTT-DTNT-DT/PC_61_BM and P3HT/PTT-DTNT-DT. At the same time, the complementary electron donor PTT-DTNT-DT can absorb more solar lights from longer wavelength, resulting in more excitons and free charge carriers. In addition, PTT-DTNT-DT can be used as hole relay to promote hole extraction from PC_61_BM to P3HT after photo excitation and charge separation [28]. In order to further confirm the charge transfer between P3HT and PTT-DTNT-DT, a series of devices based on P3HT:PTT-DTNT-DT (1:0, 1:0.2, 1:0.5, 0:1) without PC_61_BM had been fabricated. The corresponding *J*-*V* curves were shown in Figure S1. Compared with the devices only with P3HT or PTT-DTNT-DT, the cells with P3HT:PTT-DTNT-DT served as active layers exhibited higher *J*_SC_. It is indicated that excitons can be separated into free carriers at the interfaces of P3HT/PTT-DTNT-DT and then collected by external circuits even with a small amount of PTT-DTNT-DT. The excitons can only be separated at the P3HT/PC_61_BM interfaces in binary cells. However, when doping PTT-DTNT-DT into binary P3HT:PC_61_BM system, excitons dissociation of ternary devices could occur at P3HT/PTT-DTNT-DT, P3HT/PC_61_BM and PTT-DTNT-DT/PC_61_BM interfaces due to the process of charge transfer between P3HT and PTT-DTNT-DT. At the same time, Figure 4b show a quenching of the fluorescence intensity of the blends, ascribable to the photoinduced charge separation at the P3HT/PTT-DTNT-DT interface, demonstrating another possible energy transfer route is Förster resonance energy transfer (FRET) of the P3HT excitation to PTT-DTNT-DT [29,30,31]. The increase of excitons separation leads to the increase of *FF* and *J*_SC_ in ternary OPVs.

**Figure 5:**
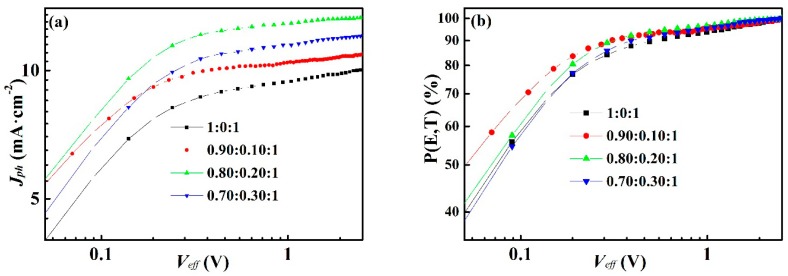
(**a**) The *J_ph_* − *V_eff_* curves and (**b**) *P*(*E*,*T*)*-V_eff_* curves of the OPVs with different PTT-DTNT-DT doping concentration.

To figure out the effect of PTT-DTNT-DT on the process of exciton generation and separation, the relationship curves between photocurrent (*J_ph_*) and effective applied voltage *(V_eff_*) of binary and ternary OPVs are showed in Figure 5a. The parameters are listed in Table S1. Here, *J_ph_* = *J_L_*− *J_d_*, *J_L_* and *J_d_* are the current under illumination and in dark, respectively. *V_eff_* = *V* − *V_a_*, *V*_0_ is voltage at *J_ph_* = 0, and *V_a_* is the applied voltage. The saturated current density (*J_sat_*) values of devices with PTT-DTNT-DT doping concentration of 0, 10, 20 and 30 wt % are 10.04, 10.94, 13.32 and 12.04 mA·cm^−2^, respectively, The increase in *J_sat_* is due to more efficient carrier transmission and collection the maximum exciton generation rate (*G*_max_) can be calculated by *J_sat_* = qL*G*_max_, where q is basic charge and L is BHJ layer thickness [32,33]. The *G*_max_ of the ternary OPVs doping 20 wt % PTT-DTNT-DT is 5.20 × 10^27^ m^−3^·s^−1^, which is larger than that of 0 wt % (3.92 × 10^27^ m^−3^·s^−1^), 10 wt % (4.27 × 10^27^ m^−3^·s^−1^), and 30 wt % (4.70 × 10^27^ m^−3^·s^−1^). We calculated the exciton dissociation efficiency *P*(*E*,*T*) determined by the *J_ph_*/*J_sat_* values at short circuit condition for OPVs with different PTT-DTNT-DT doping concentration. Thus, the *P*(*E*,*T*) value at any *V_eff_* can be obtained from the plot of *J_ph_*/*J_sat_* with respect to *V_eff_* as showed in Figure 5b. As a result, the *P*(*E*,*T*) values of devices with 0, 10, 20 and 30 wt % PTT-DTNT-DT content were 90.82%, 93.51%, 94.60% and 92.66%, respectively. The optimized ternary OPVs has higher *P*(*E*,*T*) values and *G*_max_ than other devices, which indicates that more exciton can be generated and more effective exciton separation and charge transportation can be obtained. It is further explained why the performance of OPVS is the best when the content of PTT-DTNT-DT is 20 wt %.

### 3.3. Charge Carrier Mobility

In order to further study the influence of different doping ratio of PTT-DTNT-DT on the charge carrier transport ability, the hole-only devices with the structure of ITO/PEDOT:PSS/BHJ layer (160 nm)/MoO_3_ (8 nm)/Ag (100 nm) have been prepared. The hole mobility can be calculated by Equation (1) [34]:

(1) $$J=\frac{9}{8}\varepsilon_{0}\varepsilon_{r}\mu \frac{{\left(V_{appl}-V_{bi}\right)}^{2}}{L^{3}}$$

*J* is the current density, *ε*_0_ is the vacuum dielectric constant, *ε_r_* is the relative dielectric constant of the BHJ layer, *μ* is the hole mobility, *L* is the BHJ layer thickness, *V_appl_* is the voltage between the two electrodes, and *V_bi_* is the built-in voltage [35]. Figure S2 shows the *J*-*V* curve of all hole-only devices. The hole mobility of device parameters are listed in Table S2. The calculated hole mobility *μ* of OPVs with different amount of PTT-DTNT-DT represented an apparent increase firstly from 1.68 × 10^−4^ cm^2^·V^−1^·s^−1^ (0 wt %) up to 5.37 × 10^−4^ cm^2^·V^−1^·s^−1^ (10 wt %) and 1.08 × 10^−3^ cm^2^·V^−1^·s^−1^ (20 wt %) and then a decrease down to 1.09 × 10^−4^ cm^2^·V^−1^·s^−1^ (30 wt %). The device with 20 wt % doping concentration of PTT-DTNT-DT has the highest hole transport ability among all the cells. The SCLC analysis shows that the addition of PTT-DTNT-DT leads to enhanced hole mobility, which is helpful to explain the better *J*_SC_ in the ternary system.

### 3.4. Blend Morphology

**Figure 6:**
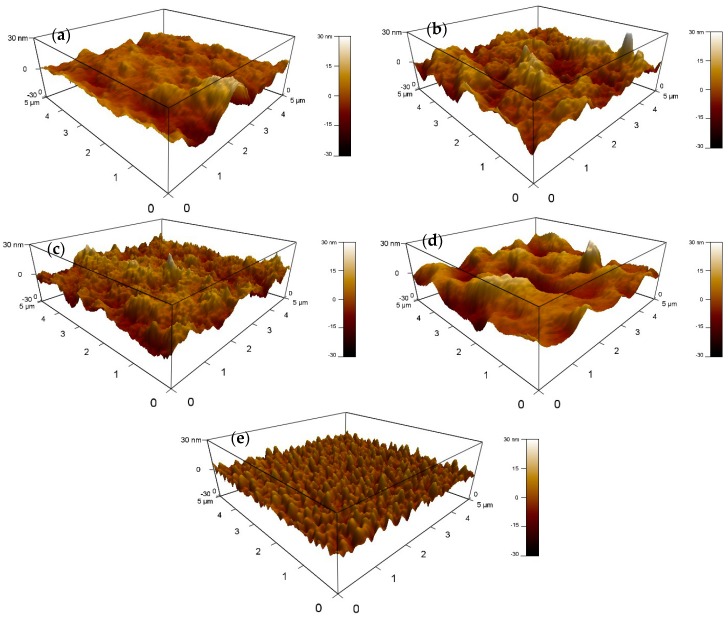
AFM 3D height images (**a**–**e**) for ternary blends of P3HT:PTT-DTNT-DT:PC_61_BM with 1:0:1, 0.90:0.10:1, 0.80:0.2:1, 0.70:0.30:1, and 0:1:1.

In order to understand the effect of PTT-DTNT-DT doping concentration on the morphology of blend films, the topography images of blend films with different PTT-DTNT-DT doping concentrations were characterized by AFM, as shown in Figure 6. The root mean square (RMS) roughness is 5.64 nm of the P3HT:PC_61_BM film. After the PTT-DTNT-DT doping concentrations of 10, 20 and 30 wt %, the RMS roughness of the blend films becomes 4.68, 4.28, and 9.23 nm, respectively. The results show that the RMS roughness of the blend films decreases first and then increases with the increasing doping concentration of PTT-DTNT-DT, which resulted from the destructed interpenetrated network. As a consequence, the films with 20 wt % PTT-DTNT-DT shows smoother surfaces than other blend films, which have an advantageous influence on exciton dissociation and charge carrier transport; therefore, the *J*_SC_ and *FF* increase obviously.

### 3.5. Impedance Spectra

Electrochemical impedance spectroscopy (EIS) is an important tool for studying charge carrier dynamics in PSCs [36,37]. The charge transport and recombination processes in the active layer were investigated by measuring the AC impedance spectra of the devices with different PTT-DTNT-DT ratios under dark conditions. Figure S3 shows the AC impedance spectroscopy results for the devices at open-circuit conditions in the frequency range of 100–1 MHz. All of the Nyquist plots of the devices can be fitted with a simple equivalent circuit as shown in the inset diagram of Figure S3. Fitting device parameters are listed in Table S3. We see that, after the PTT-DTNT-DT doping concentrations of 0, 10, 20 and 30 wt %, the CPE-P values are 0.910, 0.923, 0.947, and 0.916, respectively. Thus, the capacitance characteristics of the device with 20 wt % PTT-DTNT-DT are the closest to an ideal capacitor without defects and/or a grain boundary [38]. Simultaneously, the CPE-T increases from 1.64 × 10^−8^ to 3.56 × 10^−10^ F, suggesting that the surface of large active layer provides more charge transport pathways. However, when the PTT-DTNT-DT doping concentration increased to 30 wt %, CPE-T began to decrease, indicating that the formation of grain boundary defects would lead to charge recombination. We observed that *R*_1_ decreased to 2960 Ω of devices with the 20 wt % PTT-DTNT-DT. The large reduction of the resistance component is due to the large increase in the number of photoconductive carriers caused by light radiation in active layers. These results provide a reliable explanation for the enhancement of *J*_SC_ and *FF* of the corresponding PSCs.

### 3.6. Position of PTT-DTNT-DT

Based on the surface energy of all components, it is possible to predict the position of the single component in the ternary system [39,40]. The surface energy of copolymer and PC_61_BM was measured by contact angle measurements, as shown in the diagram of Figure S4. The surface energies of pristine P3HT, PTT-DTNT-DT and PC_61_BM are 26.09, 49.85 and 29.76 mN·m^−1^, respectively (Table S4), which were calculated by the Owens-wendt equation [41]. The calculated wetting coefficient of PTT-DTNT-DT in P3HT:PC_61_BM blends was 0.70, indicating that PTT-DTNT-DT molecules tend to reside at the interface of P3HT:PC_61_BM, which shows that doping PTT-DTNT-DT in P3HT:PC_61_BM blends makes the interfacial area increase. In this case, the D-A interface can be increased, which is favorable for the excitons to reach the interfaces and dissociate, so that the performance of ternary PSCs is improved [42].

## 4. Conclusions

A new kind of ternary system of bulk heterojunction OPVs with improved efficiency by doping PTT-DTNT-DT into conventional binary system of P3HT:PC_61_BM were successfully designed. In comparison with P3HT:PC_61_BM-based binary cells, ternary OPVs with 20 wt % PTT-DTNT-DT doping concentration demonstrated the highest PCE of 4.66%, which was 36.26% higher than that of binary cells. Assisted by AFM, EIS, SCLC and surface energy analysis, film morphology, transport, generation and recombination of carriers and the position of PTT-DTNT-DT in the blend were systematically investigated. The improvement of PCE is mainly attributed to the broadening of optical absorption, the cascade of energy levels and the optimization of the morphology of the active layer films. With the increase of the doping concentration of PTT-DTNT-DT in the donor, the properties of these OPVs increase first and then decrease, which might result in a disturbed interpenetrated network and increased charge recombination. The results show that the polymer PTT-DTNT-DT has great potential for application in OPV ternary blends because of its strong absorption in near infrared and suitable cascade energy levels with P3HT and PC_61_BM. Moreover, the ternary strategy provides a simple and effective method for the implementation of high performance OPV, and has a broad application prospect.

## Figures and Tables

**Table 1:** Photovoltaic parameters of the OPVs.

P3HT:PTT-DTNT-DT:PC_61_BM	*V*_OC_ (V)	*J*_SC_ (mA·cm^−2^)	*FF* (%)	*PCE* (%)	
				Best	Average ^2^
1:0:1	0.587 ± 0.003	9.47 ± 0.25 (9.62) ^1^	60.08 ± 0.89	3.42	3.34
0.90:0.10:1	0.576 ± 0.004	10.05 ± 0.23 (10.19) ^1^	60.97 ± 0.63	3.66	3.53
0.80:0.20:1	0.588 ± 0.002	12.44 ± 0.19 (12.68) ^1^	62.61 ± 0.78	4.66	4.58
0.70:0.30:1	0.591 ± 0.003	11.07 ± 0.09 (11.21) ^1^	57.62 ± 0.45	3.82	3.77
0:1:1	0.722 ± 0.002	10.96 ± 0.11 (11.19) ^1^	58.38 ± 0.42	4.67	4.62

^1^ The current in the parentheses is based on the EQE spectral integral. ^2^ All the parameters are based on the average of 10 devices.

## Supplementary Materials

The following are available online at http://www.mdpi.com/2073-4360/10/7/703/s1, Figure S1: *J*-*V* characteristics of cells with different weight ratios of P3HT:PTT-DTNT-DT in blend films, Figure S2: (a) *J*-*V* characteristic curves; (b) *J*^0.5^-*V* for hole-only devices with different P3HT:PTT-DTNT-DT:PC_61_BM weight ratios, Figure S3: Nyquist plots of the devices with different P3HT:PTT-DTNT-DT:PC_61_BM weight ratios. Inset shows the equivalent circuit model of the devices, Figure S4: Views of surface contact measurements of (a,d) P3HT, (b,e) PC_61_BM, and (c,f) PTT-DTNT-DT films. The measurements are carried out by using (a–c) deioninzed water and (d–f) diiodomethane as the wetting liquid, Table S1: The *G*_max_ and corresponding *J_ph_*/*J_sat_* values of the PSCs with different PTT-DTNT-DT doping ratios, Table S2: The hole mobility in different P3HT:PTT-DTNT-DT:PC_61_BM weight ratios active layers, Table S3: The Parameters employed for the fitting of the impedance spectra by use of an equivalent circuit model, Table S4: Surface energies of P3HT, PC_61_BM and PTT-DTNT-DT film.
