# Supplementary material for: 36% Enhanced Efficiency of Ternary Organic Solar Cells by Doping a NT-Based Polymer as an Electron-Cascade Donor
Source: Polymers (Basel). 2018 Jun 25;10(7):703. doi: 10.3390/polym10070703 (PMC6403668; doi:10.3390/polym10070703)
Supplement: Supplementary file 1 [file polymers-10-00703-s001.pdf]

Supplementary Information

36% enhanced efficiency of ternary organic solar cells by doping a NT-based polymer as an electron-cascade donor

Jianfeng Li[1]*, Zezhou Liang[1], Yichun Peng[2], Jie Lv[1],Xuying Ma[1],Yufei Wang[1] ,and Yangjun Xia[1]*

[1]*Key Laboratory of Optoelectronic Technology and Intelligent Control of Education Ministry, Lanzhou Jiaotong University, Lanzhou Gansu 730070, P.R.China*

[2]*School of Civil Engineering，Lanzhou Institute of Technology，Lanzhou Gansu 730050, P.R.China*

S1 Photocurrent behavior

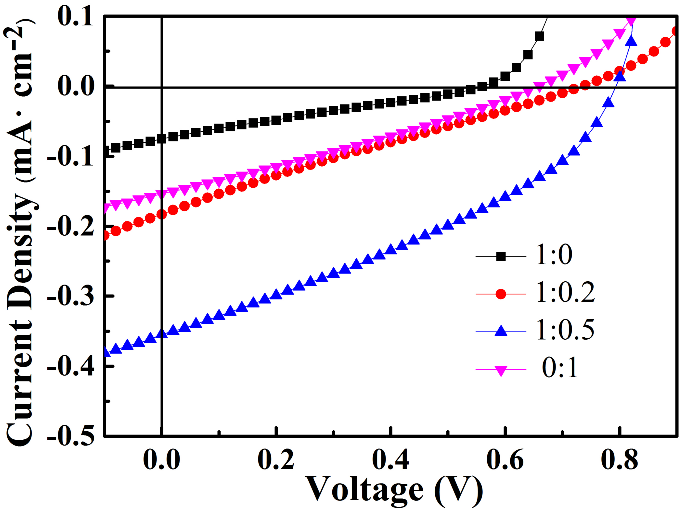

Figure S1. *J-V* characteristics of cells with different weight ratios of P3HT:PTT-DTNT-DT in blend films.

Table S1 The G_{max} and corresponding J_{ph}/J_{sat} values of the PSCs with different PTT-DTNT-DT doping ratios

P3HT: PTT-DTNT-DT:*PC₆₁BM*	J_{sat} (mA/cm^2)	G_{max} (m^{-3} s^{-1})	J_{ph}/J_{sat} (%)
1:0:1	10.04	3.92×10^{27}	90.82
0.90:0.10:1	10.94	4.27×10^{27}	93.51
0.80:0.20:1	13.32	5.20×10^{27}	94.60
0.70:0.30:1	12.04	4.70×10^{27}	92.66

S2 Estimation of hole mobility in different P3HT: PTT-DTNT-DT:$PC_{61}BM$ weight ratios active layers by SCLC model

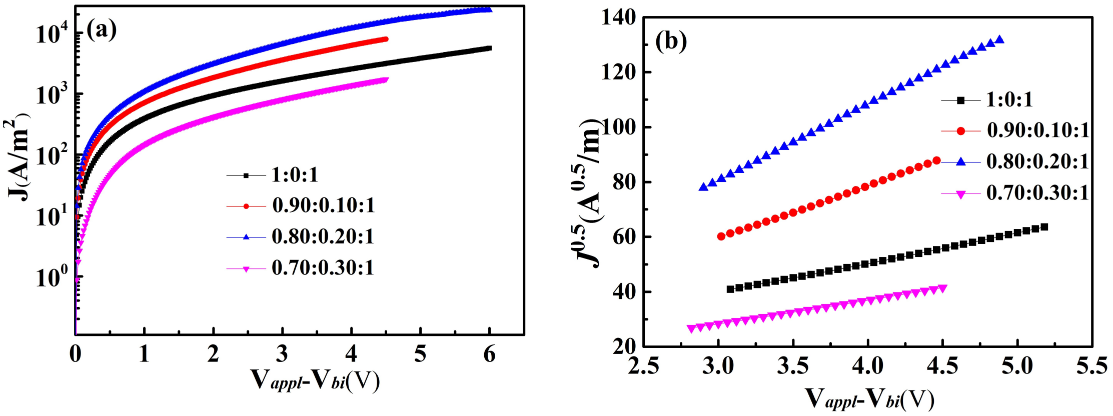

Figure S2. (a) J-V characteristic curves; **(b)** $J^{0.5}$-V for hole-only devices with different P3HT: PTT-DTNT-DT:$PC_{61}BM$ weight ratios.

Table S2. The hole mobility in different P3HT: PTT-DTNT-DT:$PC_{61}BM$ weight ratios active layers .

P3HT: PTT-DTNT-DT:$PC_{61}BM$	μ
1:0:1	1.68×10^{-4} cm^2 $V^{-1}s^{-1}$
0.90:0.10:1	5.37×10^{-4} cm^2 $V^{-1}s^{-1}$
0.80:0.20:1	1.08×10^{-3} $cm^2V^{-1}s^{-1}$
0.70:0.30:1	1.09×10^{-4} $cm^2V^{-1}s^{-1}$

S3 Electrochemical impedance spectroscopy

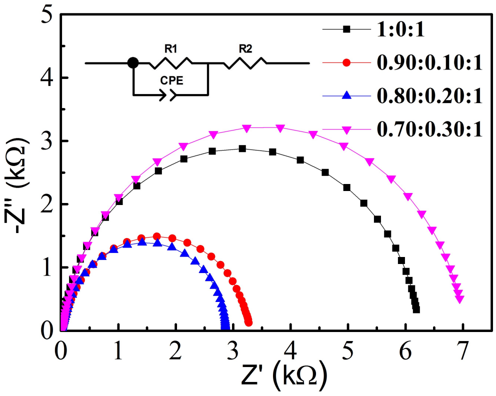

Figure S3. Nyquist plots of the devices with different P3HT: PTT-DTNT-DT:PC61BM weight ratios. Inset shows the equivalent circuit model of the devices.

Table S3 The Parameters employed for the fitting of the impedance spectra by use of an equivalent circuit model.

P3HT: PTT-DTNT-DT:$PC_{61}BM$	R_1[1](Ω)	CPE-T[2] (F/cm2)	CPE-P[3]	R_2[4] (Ω)
1:0:1	6237	1.64*10-8	0.910	27.57

0.90:0.10:1	3306	3.25*10-8	0.923	28.25
0.80:0.20:1	2960	3.56*10-8	0.947	27.27
0.70:0.30:1	7112	2.47*10-8	0.916	28.04

[1]R_1 is the resistance component forming a parallel circuit with constant phase elements.

[2]R_2 represents the series resistance.

[3]CPE-T is a capacitance

[4]CPE-P which is a non-homogeneity constant.

S4 Surface energy analysis

Table S4. Surface energies of P3HT, $PC_{61}BM$ and PTT-DTNT-DT film.

materials	Contact angle (°)		γ_d [1]	γ_p [1]	Surface energy γ
	water	diiodomethane	(mNm^{-1})	(mNm^{-1})	(mNm^{-1})
P3HT	102.91	64.69	25.67	0.42	26.09
$PC_{61}BM$	85.94	13.27	49.07	0.78	49.85
PTT-DTNT-DT	103.70	59.17	29.65	0.11	29.76

[1]γ_d and γ_p represent the surface free energies generated from the dispersion forces and the polar forces, respectively.

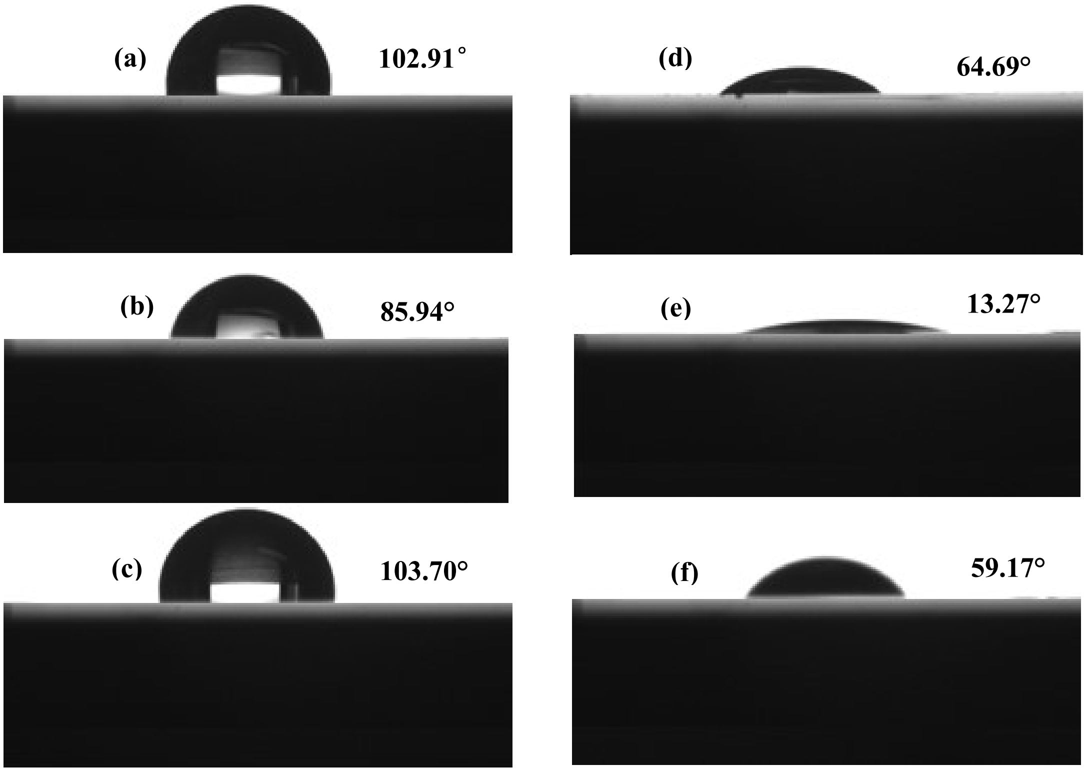

Figure S4. Views of surface contact measurements of a,d) P3HT, b,e) PC61BM, and c,f) PTT-DTNT-DT films. The measurements are carried out by using (a)–(c) deioninzed water and (d)–(f) diiodomethane as

the wetting liquid.

The interfacial surface energy (γ_{X-Y}) between X and Y in the blend films can be calculated by the equation(2) [1],

$$\gamma_{X-Y} = \gamma_X - \gamma_Y - 2\sqrt{\gamma_X \cdot \gamma_Y} \cdot e^{[-\beta(\gamma_X - \gamma_Y)^2} \quad (1)$$

Where β = 0.000115 m^4/mJ^2.

The wetting coefficient (ω_c) of a guest material C (PTT-DTNT-DT) in blends of host materials A (P3HT) and B ($PC_{61}BM$), which can predict the location of C in ternary blends, can be calculated using Young's equation(2) [2],

$$\omega_c = \frac{\gamma_{C-B} - \gamma_{C-A}}{\gamma_{A-B}} \quad (2)$$

If the wetting coefficient is larger than unity ($\omega_c > 1$), C will be located in domains of A. If $\omega_c < -1$, C will be located in domains of B. If $-1 < \omega_c < 1$, C will be located at the interface between domains of A and B.

1. Li, D.; Neumann, A.W. A reformulation of the equation of state for interfacial tensions. *J. Colloid Interface Sci.* **1990**, *137*, 304-307.
2. Sumita, M.; Sakata, K.; Asai, S.; Miyasaka, K.; Nakagawa, H. Dispersion of fillers and the electrical conductivity of polymer blends filled with carbon black. *Polym. Bull.* **1991**, *25*, 265-271.

Corresponding Authors:E-mail: ljfpyc@163.com (J. Li), xiayangjun2015@126.com (Y. Xia).
